# Digestive Proteolytic Activity in the Sunn Pest, *Eurygaster integriceps*

**DOI:** 10.1673/031.009.7001

**Published:** 2009-12-10

**Authors:** Vahid Hosseininaveh, Alireza Bandani, Fatemeh Hosseininaveh

**Affiliations:** ^1^ Department of Plant Protection, College of Horticultural Sciences and Plant Protection, University College of Agriculture and Natural Resources, University of Tehran, Karaj, Iran; ^2^ Department of Plant Protection, College of Agriculture, University of Vali-e-Asr, Rafsanjan, Iran

**Keywords:** wheat, digestive enzymes, salivary glands, proteinase

## Abstract

The Sunn pest, *Eurygaster integriceps* Puton (Heteroptera: Scutelleridae), is one of the most important pests of wheat and causes considerable damage to this valuable crop annually. Digestive proteinase activity of adult insects was investigated using general and specific substrates and inhibitors. Proteolytic activity was low when the common conventional substrates, azoalbumin, azocasein and hemoglobin were used to assay salivary glands and midguts. Using the fluorescent casein substrate (BODIPY FL casein), total proteolytic activity was measured at different pH. Maximum proteolytic activity was detected at pH 7 (100%) and 8(65%) which suggested the presence of serine proteinases in the salivary glands. There was no detectable proteolytic activity in midgut extracts. The inhibitors; PMSF (inhibitor of serine proteinases) and TPCK (a specific chymotrypsin inhibitor) showed greater than 50% inhibitory effect on total proteolytic activity, however, TLCK (specific trypsin inhibitor) and E-64(specific cysteine proteinase inhibitor) did not inhibit total proteolytic activity. Using fluorescent specific substrates for serine and cysteine proteinases (Z-Arg-AMC, Z-Arg-Arg-AMC, Z-Arg-Phe-AMC and Suc-Ala-Ala-Pro-Phe-AMZ) revealed the presence of tryptic and chymotryptic activity in the salivary gland extract. Zymogram analysis under non-reducing SDS-PAGE conditions and using the substrate APNE showed at least 8 tryptic and chymotryptic activity bands in salivary gland extracts. A single high molecular weight band with tryptic activity (165 kDa) was detected using the substrate BApNA in a zymogram analysis uisng native-PAGE. Kinetic studies showed a k_m_ value of 0.6 mM for this enzyme against the substrate BApNA .The inhibitor TLCK decreased activity of the trypsin-like enzyme up to 73% and almost completely eliminated the only band related to this proteinase in the zymogram. Soybean Kunitz type trypsin inhibitor showed no effect on proteolytic activity of the trypsin-like serine proteinase. In general, the results revealed the presence of chymotrypsin- and trypsin-like serine proteinases in the salivary gland of *E. integriceps*, and it seems that the major total proteolytic activity is due to chymotrypsin proteinases.

## Introduction

The Sunn pest, *Eurygaster integriceps* Puton (Heteroptera: Scutelleridae), is one of the most important pests of wheat in Iran and neighboring countries in the Middle and Near East (Kinaci and Kinaci 2004; Rassipour et al. 1996). The pest inserts its mouthparts into the wheat grain and then sucks the milky juices. This leads to direct damage to the yield of seeds. Besides direct reduction in yield, during feeding the injection of saliva into the grains causes protein destruction due to the saliva's hydrolytic enzymes. Wheat flour prepared from damaged seeds leads to sticky and weak dough and poor volume and texture of loaves (Kinaci and Kinaci 2004; Aja et al. 2004). This problem is caused by very specific proteinases in the saliva (Every 1993).

Chemical insecticides have been the primary means of controlling many insect pests including *E. integriceps.* However, because of many problems associated with use of synthetic pesticides in integrated pest management approaches, use of chemicals to protect grains against insect pests is limited and is being replaced by more environmentally benign alternatives (Hagstrum and Subramanyam 1996). One such approach is to enhance the resistance of important crops by plant proteins that are the plant's major defense mechanisms against herbivores. Several of these proteins are present in seeds and vegetative organs and act to regulate numbers of phytophagous insects. These compounds act on the key insect-gut digestive enzymes, the amylases and proteinases (Biggs and Mcgreagor 1996; Lawrence and Koundal 2002). Several plant proteinaceous inhibitors of insect proteinases have been identified and characterized (Garcia-Olmedo et al. 1987; Lawrence and Koundal 2002). These inhibitors are insecticidal and their function is to form complexes with digestive enzymes, which are stable and dissociate slowly. Inactivation of digestive enzymes by inhibitors results in blocking of gut proteinases that leads to poor nutrient utilization, retarded development, and death because of starvation (Jongsma and Bolter 1997; Gatehouse and Gatehouse 1999). Since there is significant variation among the properties of insect digestive enzymes, it is necessary to have more information on the gut enzymatic activities of insects to devise a rational control strategy utilizing plant-proteinaceous inhibitors (Wilhite et al. 2000).

Proteolytic activity in digestive systems of Hemiptera is due to serine and cysteine proteolytic activity in salivary glands and midgut extracts respectively (Houseman and Downe 1983; Houseman et al. 1984, 1985; Thie and Houseman 1990; Nation 2002; Zeng et al. 2002). In studies of most Hemiptera infesting wheat, attempts to develop an assay for proteolytic enzymes of digestive extracts showed no activity on several conventional substrates (Every 1993; Every et al. 2005). Studies of the digestive proteinases of wheat Hemiptera have concentrated mostly on their effect on seeds (Every and Stufkens 1999). The injected salivary proteinases remain in the grain at maturity (Cressey 1987; Every and Stufkens 1999). This enzyme specifically hydrolyses the high molecular weight glutenin subunits of gluten in the seed dough (Cressey 1987; Every 1992; Every et al. 1998). Siviri et al. (2002) showed the destructive effects of proteolytic enzymes of *E. maura* on grain of six wheat cultivars. Every et al. (2005) showed the activity of salivary proteinases from hemipteran wheat pest by relative purification and analyzed its effects on different substrates. Siviri et al. (2000) studied the pre-harvest damage done by some Hemiptera infesting wheat, especially *E. integriceps*, by evaluating the damaged wheat grains.

*E. integriceps* clearly relies on salivary gland proteinases to break down the wheat grain protein and to acquire its nitrogenous nutrients using preoral digestion. Because of complexity of gluten, it seems that the injected proteinases should be specific. As far as the authors know, there are no such studies about the pest. The aim of this study was to unravel types and properties of digestive proteinase activity of *E. integriceps* to gain a better understanding of its digestive physiology, which hopefully will lead to new strategies for management of this pest.

## Materials and Methods

### Insects

The adults of *E. integriceps* were collected from the wheat fields located in the west of Iran, Hamadan province. The samples were transferred to the Plant Protection laboratory and maintained and fed on wheat plants for 2–7 days until dissection.

### Dissection and sample preparing

The adults (800 specimens) were immobilized on ice and dissected according to Cohen (1993) under a stereoscopic microscope. The salivary glands and midguts were removed in cold distilled water separately. The collected salivary glands and the midguts (including contents) were homogenized using a hand-held glass grinder. The homogenates were then centrifuged at 16000 ×g for 5 min at 4 °C. The supernatants were freeze-dried and stored for 3 months at -20 °C. The materials were dissolved in cold distilled water when needed.

Protein concentration was determined by the method of Bradford (1976) using bovine serum albumin as standard.

### Total proteolytic activity

Total proteolytic activity of salivary gland and midgut enzyme extracts was evaluated using the conventional proteinase substrates, azoalbumin, azocasein and hemoglobin. Ten µl of enzyme extracts, solubilized in distilled water, were added to 40 µl of universal buffer composed of 40 mM sodium acetate-phosphate-borate (pH 4, 6 and 8), and 50 µl of each substrate solution (2% azoalbumin, 2% azocasein, 2% hemoglobin) was added and the mixture was incubated for 1 hour at 37 °C. The reaction was stopped by adding 100 µl of 30% TCA, held at 4 °C for 30 min and centrifuged at 10,000 × g for 15 min. In the case of azoalbumin and azocasein, a volume of the resulted supernatant was dissolved in an equal volume of 1 M NaOH before recording the absorbance at 405 nm. The TCA precipitate of the hemoglobin assay was dissolved using 1 N NaOH containing 3% Na_2_CO_3_. The 2N Folin-Ciocalteu reagent was then added to the mixture at 1N final concentration and the absorbance was recorded at 630 nm. Appropriate blanks in which TCA was added first to the substrate were prepared for each assay. Bovine trypsin (1 µg/ml) was used as positive control in all experiments.

Total proteolytic activity of the salivary gland and midgut enzyme extracts was also estimated using the substrate BODIPY FL casein (EnzChek Protease Assay Kit, http://invitrogen.com) as the fluorometric proteinase substrate at a final concentration of 0.5 µl/ml in a total volume of 100 µl using a range of pHs in a microtitre plate format using a Fluoroskan Ascent fluorimeter (Thermo Scientific, www.thermo.com). Assays were carried out at 37 °C in the dark and fluorescence was measured after 4.5 hours with an excitation wavelength filter of 485 nm and an emission wavelength filter of 538 nm. A In a preliminary experiment no detectable proteolytic activity was found in the midgut extract, so, only the salivary gland extract was only used for further experiments. All assays were performed in triplicate.

### Specific proteolytic activity

Several specific fluorescent substrates; Z-Arg-AMC (for trypsin serine proteinases), Z-Arg-Arg-AMC (for serine and cystein proteinases), Z-Phe-Arg-AMC (for serine and cystein proteinases) and Suc-Ala-Ala-Pro-Phe-AMC (for chymotrypsin serine proteinases), were used for determining of the specific protease activity in the salivary gland extract. A reaction mixture (in triplicate) consisted of 5 µl working solution (20 mM) of the substrate, 5 µl of enzyme extract in distilled water and 90 µl of universal buffer at pH 7.0. The reaction was incubated at 30 °C for 60 min and assayed at 2 min intervals.

Trypsin activity was assayed using final concentration of 1 mM BApNA (Nα-benzoyl-L-arginine-*p*-nitroanilide) as substrate. A reaction mixture consisted of 10 µl enzyme extract, 85 µl of universal buffer with desired pH (3–11) and 5 µl of the substrate (20 mM working solution). The reaction mixture was incubated at 25 °C for 30 min. The absorbance of the resulting mixture was then measured at 405 nm using a microplate reader (Dynatech MR5000) during 48 min with 6 min intervals. The assay was performed in triplicate.

### Inhibition assays

Proteases in salivary gland enzyme extract were subjected to the effect of different inhibitors. The inhibitors used and their concentrations were: serine protease general inhibitor, 1–2 mM PMSF (phenylmethylsulfonyl fluoride); trypsin inhibitors, TLCK (Nα-p-tosyl-L-lysine chloromethyl ketone), 10 µg ml^-1^ soybean Kunitz type trypsin inhibitor; chymotrypsin inhibitors, 1 mM TPCK (N-tosyl-L-phenylalanine chloromethyl ketone), 0.1 mM chymostatin; cysteine protease inhibitor, 1 and 10 µM E-64 (L-trans-epoxysuccinyl-leucylamido-(4-guanidino)-butane), and serine and cysteine protease inhibitor, 10 µg ml^-1^ leupeptin. To determine the effect of these compounds on enzyme activities, the enzyme extracts were pre-incubated with the appropriate inhibitors for 15 min. The assays were carried out as described in the sections total proteolytic activity (fluorimetric assay) and tryptic activity. All assays were replicated three times.

### Kinetic studies

Kinetic studies were carried out for trypsin serine proteinases using 9 appropriate concentrations of the substrate BApNA (0.02 to 1 mM). The Michaelis-Menten constant (K_m_) was evaluated by non-linear regression analysis using the software package Prism (GraphPad Software, www.graphpad.com).

### Electrophoretic zymogram

Electrophoretic detection of proteolytic enzymes was carried out using a native (non-denaturing SDS-PAGE) 12.5% and 4% resolving and stacking Polyacrylamide gels, respectively (Laemmli 1970). To visualize enzymes with serine (chymotrypsin and trypsin) proteinase activity, the gel was equilibrated with 0.1 M sodium phosphate buffer, pH 7.6 (three washes of 5 min each at room temperature), then incubated in the dark at 37 °C for at least 3 hours with freshly prepared artificial substrate, N-acetyl-DL-phenylalanine α-naphtyl ester (APNE) containing fast blue running dye (Uriel and Burges 1968; Broadway 1997). After incubation, the gel was washed with distilled water and then was immersed in 2% acetic acid.

Detection of tryptic activity in the gel was performed using an overlay technique according to Vinokurov et al. (2005). After electrophoresis (native-PAGE), the gel was soaked for 15 min in the buffer (50 mM Tris-HCl, pH 8). The buffer was then removed and the gel was carefully covered by a nitrocellulose membrane (0.45 µm pore size) that had been presoaked for 40 min in the substrate solution (BApNA, 1 mg ml-1) and slightly air dried. The gel and membrane were incubated at 37 °C until faint yellow bands became visible on the membrane. The membrane was then removed and the librated pNA was diazotized by subsequent incubations of 5 min each in 0.1% sodium nitrite, 0.5% ammonium sulfamate and 0.05% N-(l-naphthyl) ethylenediamine. After pink bands of tryptic activity formed, membranes were scanned.

After tryptic enzyme was shown to be present, the effects of four main protease inhibitors; 1 mM PMSF, 0.1 mM TLCK, 0.1 mM TPCK and 10 µM E-64, were assayed for their effects on tryptic activity of the salivary gland extract. After electrophoresis, the gel was cut into different lanes and each lane was presoaked in the prepared inhibitor solutions (50 mM Tris-HCl buffer, pH 8) for 15 min. Visualization of the tryptic activity bands was done using the procedure mentioned above.

## Results

### Total proteolytic activity

**Figure 1.**
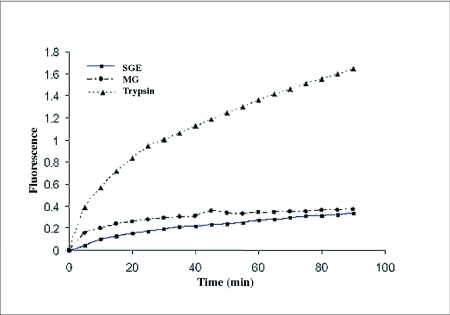
Proteolytic activities of salivary gland and midgut extracts on fluorescent substrate in comparison with positive control (trypsin) at pH 8.0 (SGE: salivary gland extract, MG: midgut).

Low levels of proteolytic activity were detected in salivary gland and midgut enzyme extracts when azocasein, azoalbumin and hemoglobin were used as the substrate (Table 1). The preliminary experiment using the fluorescent substrate showed detectable proteolytic activity in the salivary gland extract and low levels of activity in the midgut enzyme extract (Figure 1).

**Figure 2.**
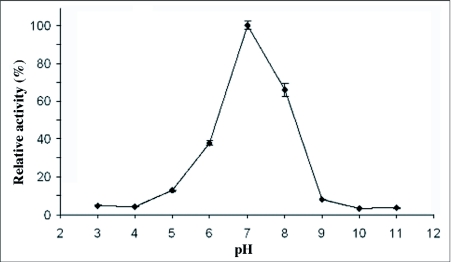
Proteolytic activity of the salivary gland extracts at different pHs using the fluorescent casein substrate.

Proteolytic activity of the salivary gland homogenate was considerable at pH 6.0–8.0 and maximal proteolytic activity occurred at pH 7.0 using the substrate BODIPY FL casein (Figure 2). The proteolytic activity was lower than 15% of maximal activity at pH outside of 6.0–8.0.

**Table 1.**
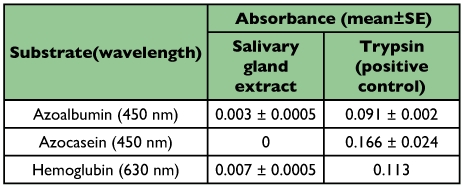
Proteolytic activity of salivary gland enzyme extract on conventional substrates

### Specific proteolytic activity

**Figure 3.**
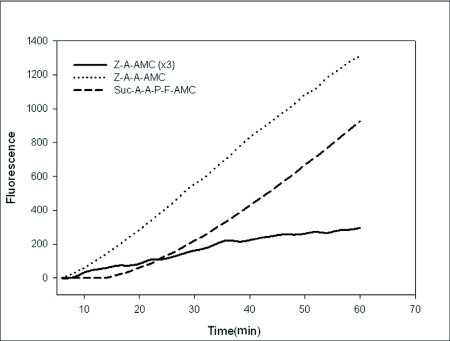
Hydrolytic activity of the salivary gland extract on several specific fluorescent substrates.

Maximal specific proteinases activity of the salivary gland extract was attained with the substrate Z-Arg-Arg-AMC with the initial velocity of 26 at 355 nm/min (Figure 3). The initial velocities of activity of salivary gland extract on the substrates Z-Arg-AMC and Suc-Ala-Ala-Pro-Phe-AMC were obtained as 5 and 22 at 355 nm/min respectively. No proteolytic activity was obtained using the substrate Z-Phe-Arg-AMC.

**Figure 4.**
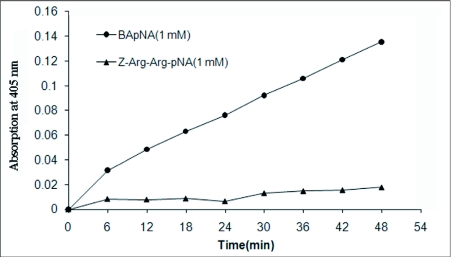
Hydrolytic activity of the salivary gland extract on trypsin specific substrates.

**Figure 5.**
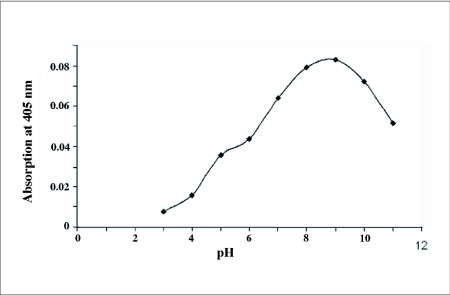
Tryptic activity of the salivary gland extract as affected by pH.

Specific trypsin substrate (BApNA) was hydrolyzed faster (0.0027 OD at 405 nm/min) than the other specific trypsin substrate, Z-Arg-Arg-pNA (0.0003 OD at 405/min) (Figure 4). No considerable tryptic activity was detected using the substrate Z-Phe-Arg-pNA. Salivary gland extracts showed an optimum tryptic activity against the specific trypsin substrate BApNA at alkaline pH broad optimum at the range 7.0–11.0 (Figure 5). Maximal activity was obtained at the pH 9.0.

### Inhibition assays

**Figure 6.**
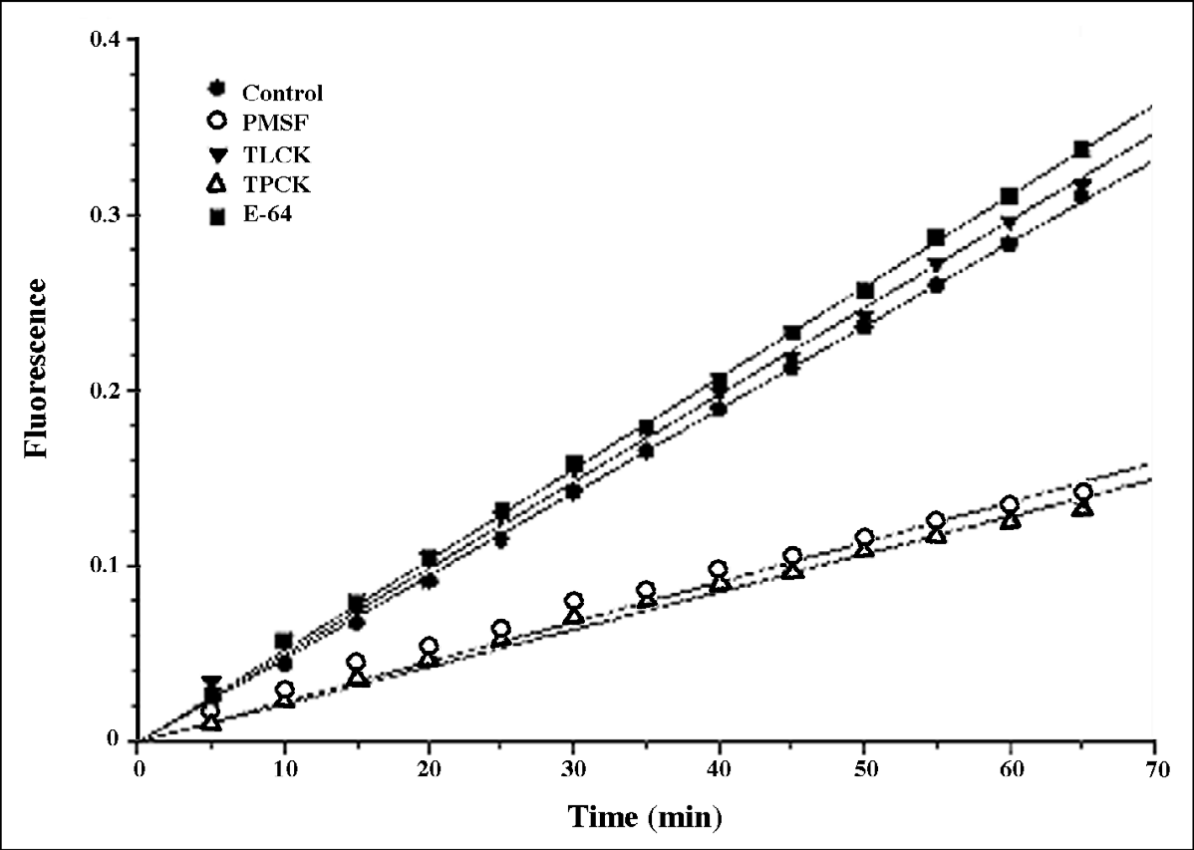
Effect of four main proteinase inhibitors on total proteolytic activity of the salivary gland extract against the fluorescent casein substrate.

The total proteolytic activity of the salivary gland extract was further characterized using the specific proteinase inhibitors, PMSF (general serine proteinase inhibitor) and TPCK (chymotrypsin serine proteinase) (Figure 6). They showed significant 51 and 55% decreases in total proteolytic activity, respectively, suggesting chymotryptic serine proteinase activity in the salivary gland extracts. No significant inhibition was obtained when the inhibitors E-64 (cysteine proteinase inhibitor) and TLCK (trypsin serine proteinase) were used suggesting that there was no cysteine proteinase activity in the salivary gland extract.

The inhibitors PMSF, soybean Kunitz type trypsin inhibitor (SKTI) and E-64, at their appropriate concentration, didn't show any inhibitory effect on tryptic activity of the salivary gland extract against the substrate BApNA (Table 2). The inhibitor E-64 showed just 5.4% inhibitory effect at the highest recommended concentration. The most effective inhibitors of tryptic activity, TLCK and leupeptin (serine and cysteine proteinase inhibitors) showed 78 and 73% inhibition, respectively. A low inhibition by the chymostatin inhibitor was also observed on tryptic activity (24% decrease in tryptic activity).

### Kinetic studies

**Figure 7.**
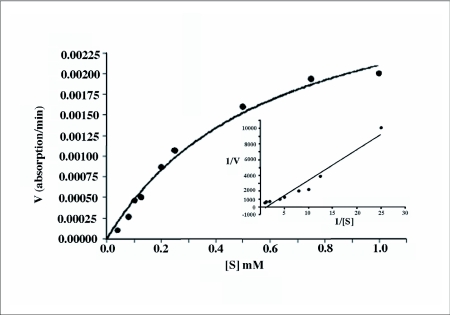
Michaelis-Menten and Lineweaver-Burk plots of tryptic activity of the salivary gland extract on substrate BApNA.

The K_m_ value of trypsin serine proteinase determined for BApNA substrate was 0.6±0.06 mM (Figure 7). The estimated k_m_ value is similar to the previously reported k_m_ in the other insects (0.08–0.93 mM) (Tsybina et al. 2005).

### Zymogram studies

**Figure 8.**
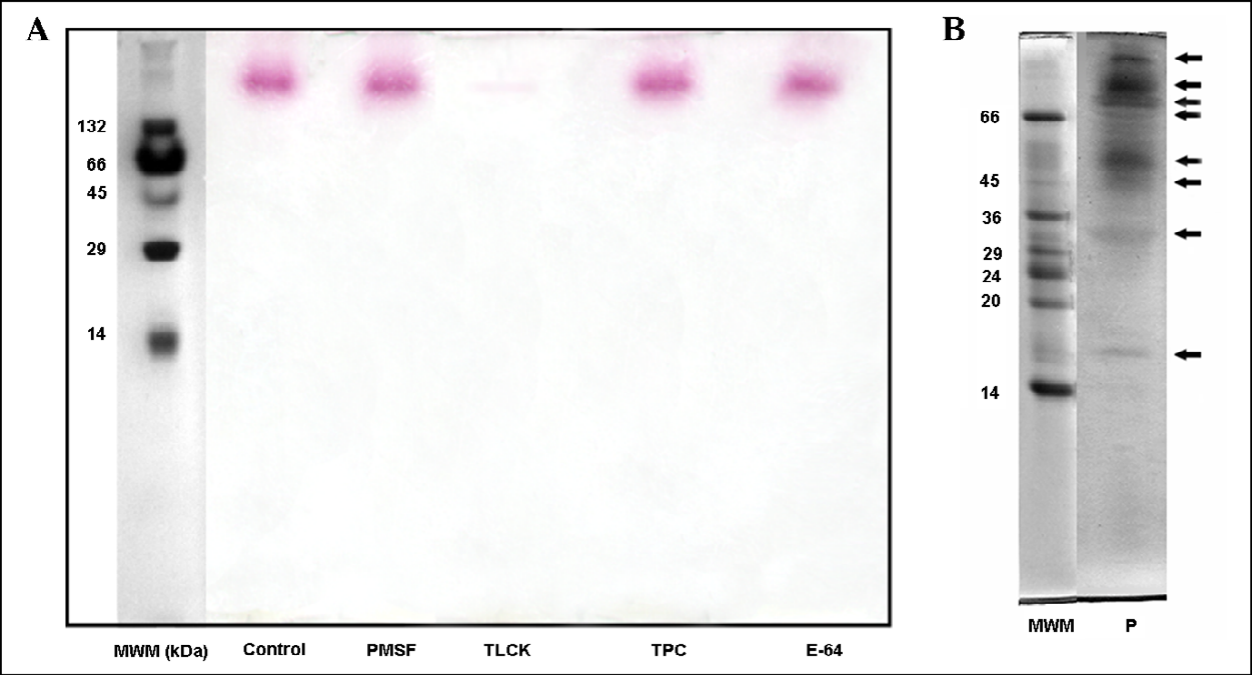
(A) Zymogram analysis of the effect of some proteinase inhibitors on BApNA hydrolytic activity of salivary gland extract (MWM: molecular weight markers, Control: no inhibitor, PMSF: 1 mM, TLCK: 0.1 mM, TPCK: 0.1 mM and E-64: 10 µM). (B) Zymogram analysis of hydrolytic activity of the salivary gland extract on the substrate APNE (MWM: molecular weight markers in kDa, P: bands with proteolytic activity).

Further characterizations of the proteinase activity of salivary gland extract from *E. integriceps* adults using substrate non-denaturing SDS-PAGE are shown in Figures 8A, B. Zymogram analysis using the substrate N-acetyl-DL-phenylalanine β-naphtyl ester (APNE) revealed at least eight bands with chymotryptic and tryptic activity (16–81 kDa).

Applying the substrate BApNA after electrophoresis in the overlay technique for detecting tryptic activity in the salivary gland extract visualizes only a single band with tryptic activity. The molecular weight of this band was estimated as 165 kDa. The inhibitor TLCK caused complete inhibition of the trypsin-like activity in the zymogram study using the substrate BApNA. The inhibitory effect of the other inhibitors (PMSF, TPCK and E-64) could not be detected in the gel electrophoresis zymogram.

## Discussion

The substrates azoalbumin, azocasein and hemoglobin are routinely used as conventional substrates for detecting total proteolytic activity in many cases (Cohen 1993; Elpidina et al. 2001). Our data show that the proteinases in salivary gland and midgut extracts from *E. integriceps* adults are not capable of hydrolyzing these substrates. This situation is also the case in other Hemiptera infesting wheat. Assaying digestive proteinase from the hemipteran, *Nysius huttoni*, is not possible using common proteinase substrates (Every 1991, 1993; Every et al. 2005). Its digestive proteinases don't work on a wide range of the substrates such as gelatin, hemoglobin and bovine serum albumin. This is because of high specificity of the digestive proteinases for hydrolyzing wheat protein (Every 1993). Therefore, special and non-conventional methods have been applied for detecting digestive proteolytic activity in Hemiptera infesting wheat (Every 1991, 1993). It seems that this situation is also the in case for *E. integriceps.*

Despite non-detectable proteolytic activity in the salivary gland and midgut extracts from *E. integriceps* using common substrates, salivary gland extracts showed hydrolytic activity against the fluorescent casein substrate after long enzymatic incubation time (4.5 hours). A comparison of two classes of substrates revealed that fluorescent casein assay (fluorimetric detection) is more sensitive than the common substrate assay (spectrophotometery). Optimal proteolytic activity of the salivary gland extract was obtained at slightly acidic to slightly alkaline pH suggesting there was cysteine and serine proteinase activity in the extract. It seems that serine proteinases are the major hydrolyzing enzyme in the salivary gland extract from *E. integriceps* because their greater activity at alkaline pH (66%) in comparison with acidic pH (38%). Investigations of other Hemiptera also show that the serine proteases are the prevailing proteolytic enzymes in their saliva according to pH optimum of their activity, specific substrates and inhibition assays (Laurema et al. 1985; Cohen 1993; Colebatch et al. 2002). At different pH, proteolytic activity occurs with its maximal activity at a single point (pH 7.0) suggesting there are not complicated proteolytic enzymes in the salivary gland extract.

Specific trypsin serine proteinase did not contribute to total proteolytic activity, whereas chymotrypsin serine proteinase activity did contribute to total proteolytic activity. It seems that the trypsin proteinase is a specific serine proteinase because there was no inhibition by a common serine proteinase inhibitor (PMSF) but a specific trypsin serine proteinase (TLCK) was effective. So, the resultant total proteolytic activity is mainly due to chymotrypsin serine proteinases. The only detected tryptic band using zymogram analysis was a single high molecular weight band. A similar high molecular weight proteinase has been reported in some insects (Bell et al. 2005). Zymogram analysis using APNE as the substrate also revealed chymotryptic activity in the salivary gland. Trypsin serine proteinases are able to hydrolyze the substrate APNE. So the bands detected in the gel are mainly chymotrypsins but also trypsin proteinases.

**Table 2.**
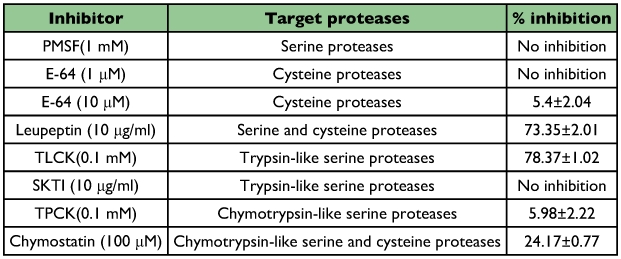
Effect of some inhibitors on tryptic activity (using BApNA as substrate) of salivary gland extract.

Serine proteinases are the major proteases found in the salivary gland of many heteropteran insects (Cohen 1993; Zeng 2002). The present study also indicates that chymotrypsin and trypsin serine proteinases are the main endopeptidases in the salivary gland of *E. integriceps.* In other pests feeding on wheat grain, such as *Trogoderma grananum*, trypsin and chymotrypsin serine proteinases have been shown to be the main endopeptidases in midgut extracts (Hosseininaveh et al. 2007). However, it seems that the chymotrypsin serine proteinases are more general than trypsin serine proteinases in salivary glands of *E. integriceps.* Trypsin serine proteinases in the salivary gland are more specific than the chymotrypsin serine proteinases.

## Abbreviations

**APNE**: N-acetyl-DL-phenylalanine α-naphtyl ester,
**BApNA**: Nα-benzoyl-L-arginine-p-nitroanilide,
E-64: cysteine proteinase inhibitor,
**leupeptin**: serine and cysteine protease inhibitor,
**PMSF**: serine proteinase inhibitor,
**SKTI**: serine protease inhibitor,
**TLCK**: trypsin inhibitor,
**TPCK**: chymotrypsin inhibitor
